# One-pot synthesis of monodisperse CoFe_2_O_4_@Ag core-shell nanoparticles and their characterization

**DOI:** 10.1186/s11671-018-2544-z

**Published:** 2018-06-08

**Authors:** Shuta Hara, Jumpei Aisu, Masahiro Kato, Takashige Aono, Kosuke Sugawa, Kouichi Takase, Joe Otsuki, Shigeru Shimizu, Hiroki Ikake

**Affiliations:** 1 Department of Materials and Applied Chemistry, College of Science and Technology, Nihon University, 1-8-14 Kandasurugadai, Chiyoda-ku, Tokyo, 101-8308 Japan; 2 Department of Physics, College of Science and Technology, Nihon University, 1-8-14 Kandasurugadai, Chiyoda-ku, Tokyo, 101-8308 Japan

**Keywords:** Core-shell nanoparticles, Cobalt ferrite, Supermagnetism, Surface plasmon resonance

## Abstract

In recent years, monodispersed magnetic nanoparticles with a core/shell structure are expected for their wide applications including magnetic fluid, recoverable catalysts, and biological analysis. However, their synthesis method needs numerous processes such as solvent substitution, exchange of protective agents, and centrifugation. A simple and rapid method for the synthesis of monodispersed core-shell nanoparticles makes it possible to accelerate their further applications. This paper describes a simple and rapid one-pot synthesis of core (CoFe_2_O_4_)-shell (Ag) nanoparticles with high monodispersity. The synthesized nanoparticles showed plasmonic light absorption owing to the Ag shell. Moreover, the magnetic property of the nanoparticles had a soft magnetic behavior at room temperature and a hard magnetic behavior at 5 K. In addition, the nanoparticles showed high monodispersity with a low polydispersity index (PDI) value of 0.083 in hexane.

**Electronic supplementary material:**

The online version of this article (10.1186/s11671-018-2544-z) contains supplementary material, which is available to authorized users.

## Background

Over the last decade, magnetic nanoparticles with a core/shell structure have gained a lot of attention in a wide range of fields from engineering to medical sciences owing to the applications of magnetic fluids [1, 2], magnetic separation [1–3], recoverable catalysts [1, 2, 4–7], drug delivery system [1, 8–10], and an enhanced magnetic resonance imaging (MRI) contrast agents [7, 9–11].

Among the magnetic nanoparticles, a spinel ferrite nanoparticle has frequently been employed as a magnetic core because of its excellent magnetic and electrical properties [12]. Particularly, cobalt ferrite (CoFe_2_O_4_) nanoparticles have a large maximum coercive field (*H*_c_), even with a small size as well as a remarkable chemical stability and a mechanical hardness [13–17]. Although many different chemical methods have been developed to fabricate CoFe2O4 nanoparticles, the thermal decomposition method has recently been employed one of the most promising procedures to obtain highly, structurally, and morphologically controlled nanoparticles with a high crystallinity [13, 17, 18].

Magnetic nanoparticles with a core/shell structure have attracted a great deal of attention due to their multifunctionality including optical, electronic, and magnetic properties [6, 8, 10, 19]. In particular, the Au shell-coated magnetic nanoparticles have widely been studied in order to provide not only the surface plasmon properties but also a reactive surface for strong binding to organic compounds containing thiol groups [3, 20]. Typically, an approach of combined two-step thermal decomposition process can continuously synthesize from cores to shells, resulting in the formation of Au-coated magnetic nanoparticles with a high monodispersity [20]. On the other hand, Ag shell-coated magnetic nanoparticles have not been synthesized by this approach in spite of excellent plasmonic properties, a higher extinction coefficient, a sharper extinction band, a higher light scattering-to-extinction effect, and strong local electromagnetic fields of Ag shells.

In this study, we succeeded in synthesizing Ag shell-coated CoFe_2_O_4_ nanoparticles by a simple and rapid one-pot method involving two thermal decomposition processes. It was confirmed that our synthesized nanoparticles formed a precise core-shell structure, as compared with those synthesized in a previous paper [21, 22]. In addition, we demonstrated that the CoFe_2_O_4_@Ag showed the localized surface plasmon resonance (LSPR) originated from the Ag shells. In the investigation of the magnetic property, this core-shell nanoparticle revealed soft magnetic behavior with *H*_c_ of 70 Oe at 300 k and hard magnetic behavior with 11 k Oe at 5 K.

## Method/Experimental

### Material

Fe(acac)_3_ and Co(acac)_2_ were purchased from Tokyo Chemical Industry. Diphenyl ether, oleylamine (OAm), and silver(I) acetate were purchased from Wako. Oleic acid (OA) was purchased from Kanto Chemical.

### Synthesis of CoFe2O4@Ag

**Scheme 1:**
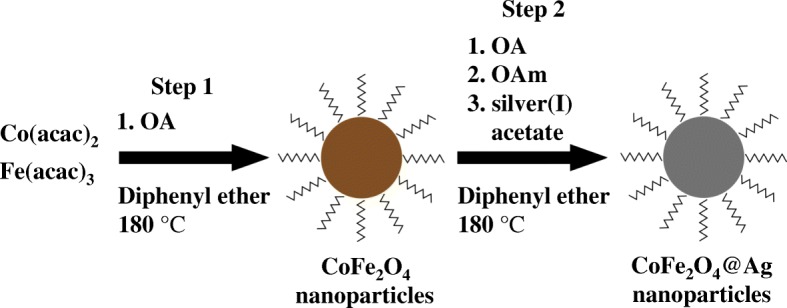
Procedure for synthesizing CoFe_2_O_4_@Ag nanoparticles

The CoFe2O4@Ag were synthesized by the two-step high thermal decomposition method (Scheme 1). Fe(acac)_3_ (0.353 g, 1 mmol), Co(acac)_2_ (0.129 g, 0.5 mmol), and OA (3.39 g, 12 mmol) were dissolved in 30 mL of diphenyl ether, which was pre-treated by heating at 180 °C for 30 min. A mixture was heated at 180 °C for 16 h under vigorous stirring. The solution color gradually turned from dark red to fine black. After cooling at room temperature, a mixture of OA (1.48 g, 5.2 mmol), OAm (8.13 g, 30.4 mmol), and silver acetate (0.61 g, 3.6 mmol) dissolved in 100 mL of diphenyl ether was added to the mixture, followed by heating at 180 °C for 1.5 h. The color of the mixture further turned to metallic dark purple during heating. After cooling, 400 mL of methanol as a poor solvent was added to the mixture solution, followed by centrifugation (5000 rpm, 5 min) and the redispersion in 60 mL of hexane. Although the nanoparticles dispersed in the solution might be able to be magnetically separated, it takes time to recover. The centrifugation process was repeated several times to remove the unreacted precursors. Finally, by centrifuging the colloidal hexane solution (14,000 rpm, 20 min), the resulting precipitates were removed. The net weight of nanoparticles by this method is about 60 mg as 1 mg/mL of the colloidal hexane solution. The CoFe_2_O_4_ nanoparticles as a reference were prepared by performing only step 1 in Scheme 1.

### Characterization and Calculation

The morphology of nanoparticles was observed using field-emission transmission electron microscopy (TEM) (Hitachi, Ltd., FE 2000). The crystal structures were measured with X-ray diffraction (XRD) (PANalytical, X’Pert PRO MPD) in the range of 2*θ* = 20° to 80° by using the CuK α-ray. Element composition of nanoparticles was analyzed by X-ray photoelectron spectroscopy (XPS) (KARATOS ESCA 3400). Etching operation was performed with Ar ion gun. The magnetization measurements were performed by a superconducting quantum interference device (SQUID) (Cryogenic, S700X-R). The optical properties were measured on a UV-visible spectrophotometer (Jasco, V-670). Dynamic light scattering (DLS) (Malvern, zetasizer-nano-zs) was measured with 633-nm laser line. For the optical properties of our synthesized core-shell nanoparticles, the experimental data are supported by Mie scattering calculations which were carried out by Bohren and Huffman’s solution [23] using the MATLAB code written by Mätzler [24]. Dielectric functions for the Ag were taken from Reference [25].

## Results and Discussion

**Fig. 1:**
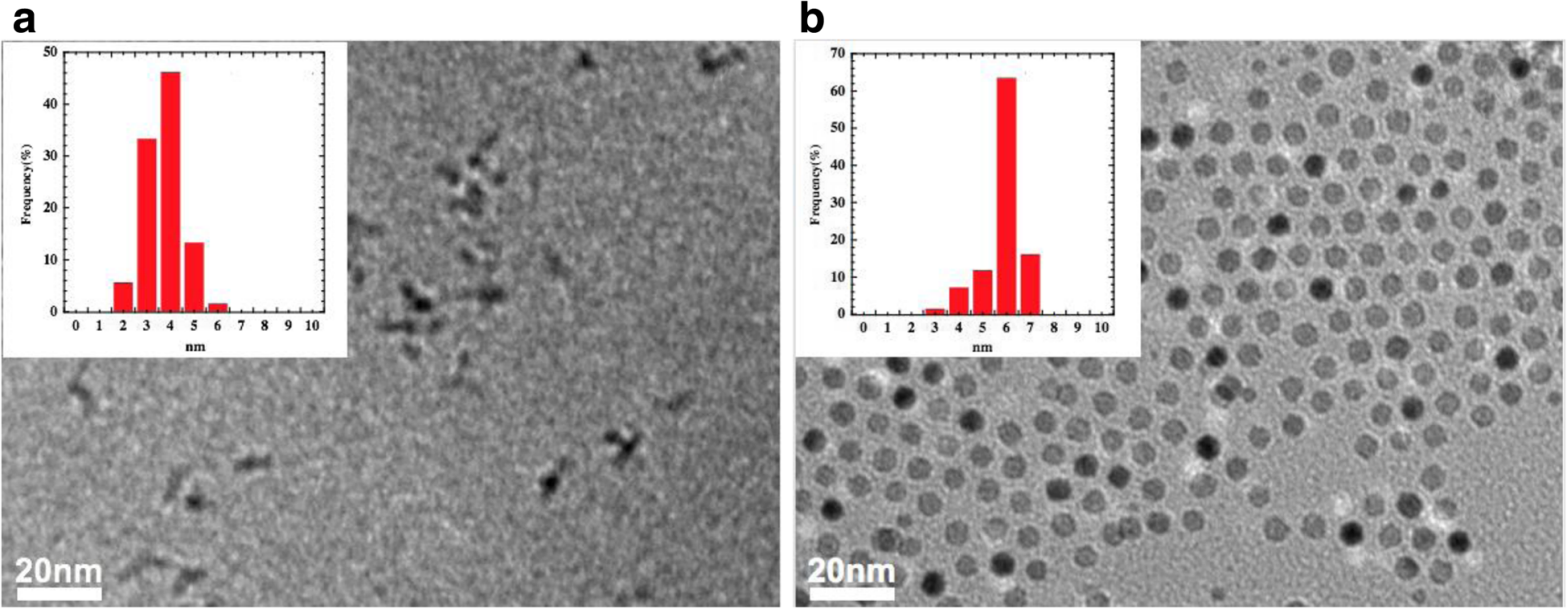
TEM images and particle size histograms for nanoparticles of **a** CoFe_2_O_4_ and **b** CoFe_2_O_4_@Ag

Figure 1 shows the TEM images of CoFe_2_O_4_ nanoparticles and CoFe_2_O_4_@Ag core-shell nanoparticles. As shown in the insets of Fig. 1, the size distributions of both nanoparticles are narrow. The average sizes (mean ± S.D.) of them are 3.5 ± 0.76 and 5.5 ± 0.77 nm, respectively. From these results, the thickness of Ag shell was estimated to be ca. 1 nm. Aggregation of CoFe_2_O_4_ particles occurred but not for CoFe_2_O_4_@Ag nanoparticles. This is possibly due to a higher surface energy of the CoFe2O4 nanoparticles than that of the CoFe_2_O_4_@Ag nanoparticles because of a larger surface-to-volume ratio of the CoFe_2_O_4_ nanoparticles [26]. Also, residual CoFe_2_O_4_ nanoparticles (cores) could not be observed in the sample of CoFe_2_O_4_@Ag. This result suggests that almost all the cores are uniformly coated with the silver Ag shell.

**Fig. 2:**
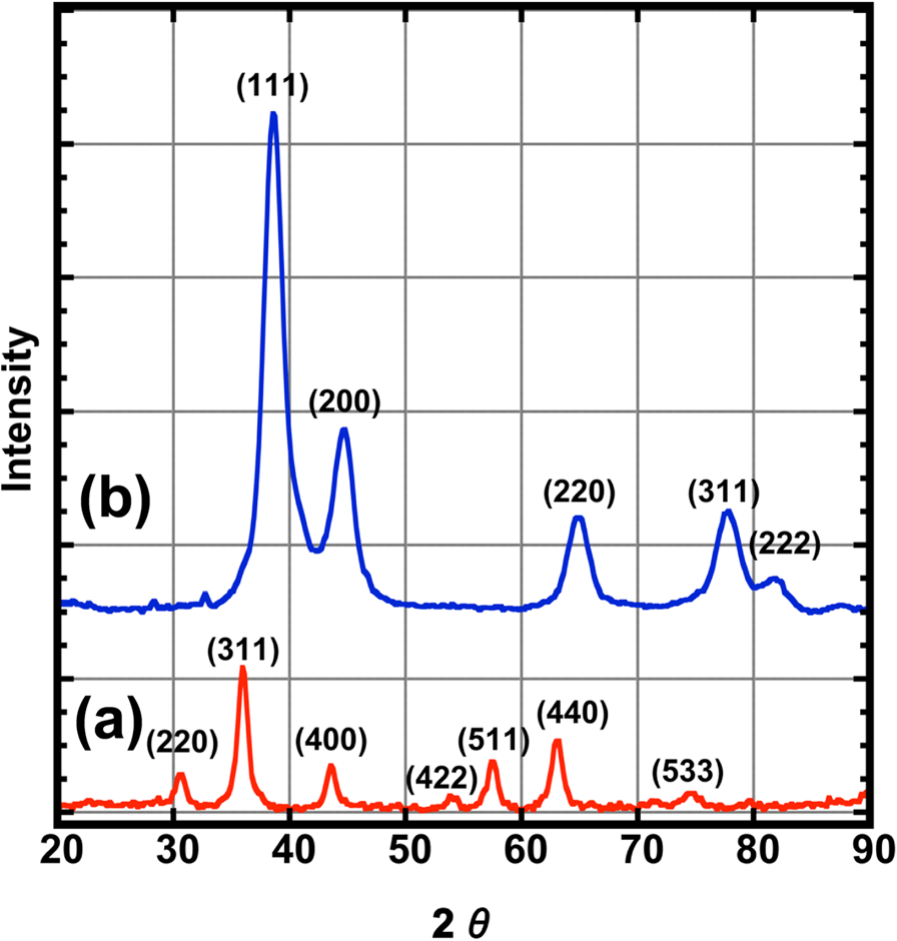
XRD pattern of for nanoparticles, (**a**) CoFe_2_O_4_ (red line) and (**b**) CoFe_2_O_4_ @Ag (blue line)

Figure 2 presents the XRD patterns for the CoFe_2_O_4_ and the CoFe_2_O_4_@Ag nanoparticles. The diffraction peaks of CoFe_2_O_4_ nanoparticles at 2*θ* = 30.50°, 35.75°, 43.50°, 53.8°, 57.5°, 63.0°, and 74.4° show the formation of a single crystallographic phase, which can be indexed as the cubic structure of spinel oxides [17]. On the other hand, the diffraction peaks of CoFe_2_O_4_@Ag at 2*θ* = 38.42°, 44.50°, 64.91°, 77.75°, and 81.83° correspond to those of the standard face-centered cubic (fcc) phase of Ag [10]. The intensity of the diffraction peaks of CoFe_2_O_4_ are relatively weak, and its main peak overlaps with Ag; therefore, all emerge into those of Ag. The crystallite size was calculated from the full width at half maximum (FWHM) of the highest intensity diffraction peak, which is based on the Debye-Scherrer equation,

1 $$ t=0.9l/b\ \cos\ y $$

where *t* is the crystallite size, *l* is the wavelength of Cu-Ka radiation, *b* is the FWHM, and *y* is the diffraction angle of the strongest peak. The crystal sizes evaluated from the diffraction patterns were 7.1 and 3.6 nm for CoFe_2_O_4_ nanoparticles and CoFe_2_O_4_@Ag nanoparticles, respectively. The crystal size of CoFe_2_O_4_ nanoparticles was observed to be larger than the size of TEM because of the residue of CoFe_2_O_4_ nanoparticles out of size distribution, which could not be removed by centrifugation in hexane. On the other hand, the crystal size from XRD showed an agreement in CoFe2O4@Ag nanoparticles considering that the crystal size of Ag shell has to be smaller than the size of TEM. The size of the colloid after the silver coating reaction enables to select by centrifugation due to its heavyweight in hexane.

**Fig. 3:**
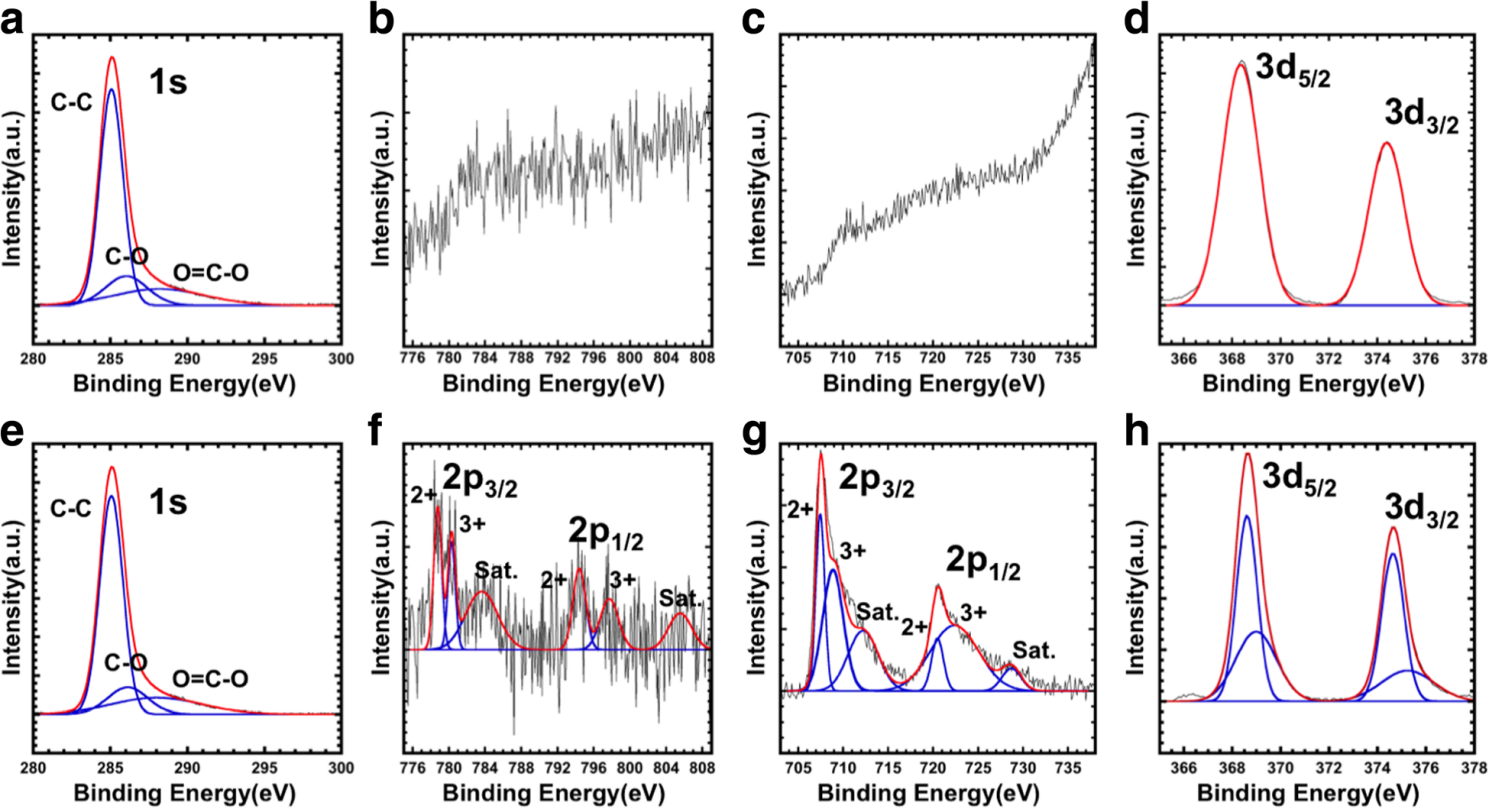
XPS spectra of CoFe_2_O_4_@Ag by argon ion etching before (**a**–**d**) and after (**e**–**h**). **a**, **e** C 1 s. **b**, **f** Co 2p. **c**, **g** Fe 2p. **d**, **h** Ag 3d

To evaluate the internal composition of the obtained nanoparticles with a core-shell structure, the nanoparticle surfaces were etched using Ar ion gun in the chamber [27]. According to the previous studies, when the particles had a precise core-shell structure, the peak intensity of the element contained in the core should be increased as the etching progresses. As shown in Fig. 3a–d, to determine the surface composition of CoFe_2_O_4_@Ag nanoparticles, we measured the XPS spectra before the Ar ion etching. In the initial surfaces, the peek C (1 s) were easily observed in nanoparticles due to the presence of the protective agent on the surface of the nanoparticles (Fig. 3a). The spectrum of C (1 s) was decomposed, and a peak derived from C-O-C was observed, which is derived from oleic acid modified on the surface. While the peaks of Ag(3d) were observed, those of Fe(2p) and Co(2p) could not be observed, indicating that the core was completely covered with the Ag shells (Fig. 3b–d). On the other hand, the peaks of Fe(2p) and Co(2p) were observed in the nanoparticles after the etching operation with argon ion (Fig. 3f, g). The peaks of Fe(2p) and Co(2p) are decomposed and can be assigned to Fe^2+^, Fe^3+^, Co^2+^, and Co^3+^, respectively. The formation of both types of charge carriers results from the loss of oxygen during the high-temperature reaction process [28, 29]. For the charge compensation, a part of Fe3+ is converted to Fe2+, and a part of Co2+ is converted to Co3+. Furthermore, each of the Ag(3d) peak after the etching can be decomposed into two peaks (Fig. 3h), due to the difference in electronic state at between the nanoparticle surfaces and the inside of the shells. These results indicate that the precise core-shell structure is formed.

**Fig. 4:**
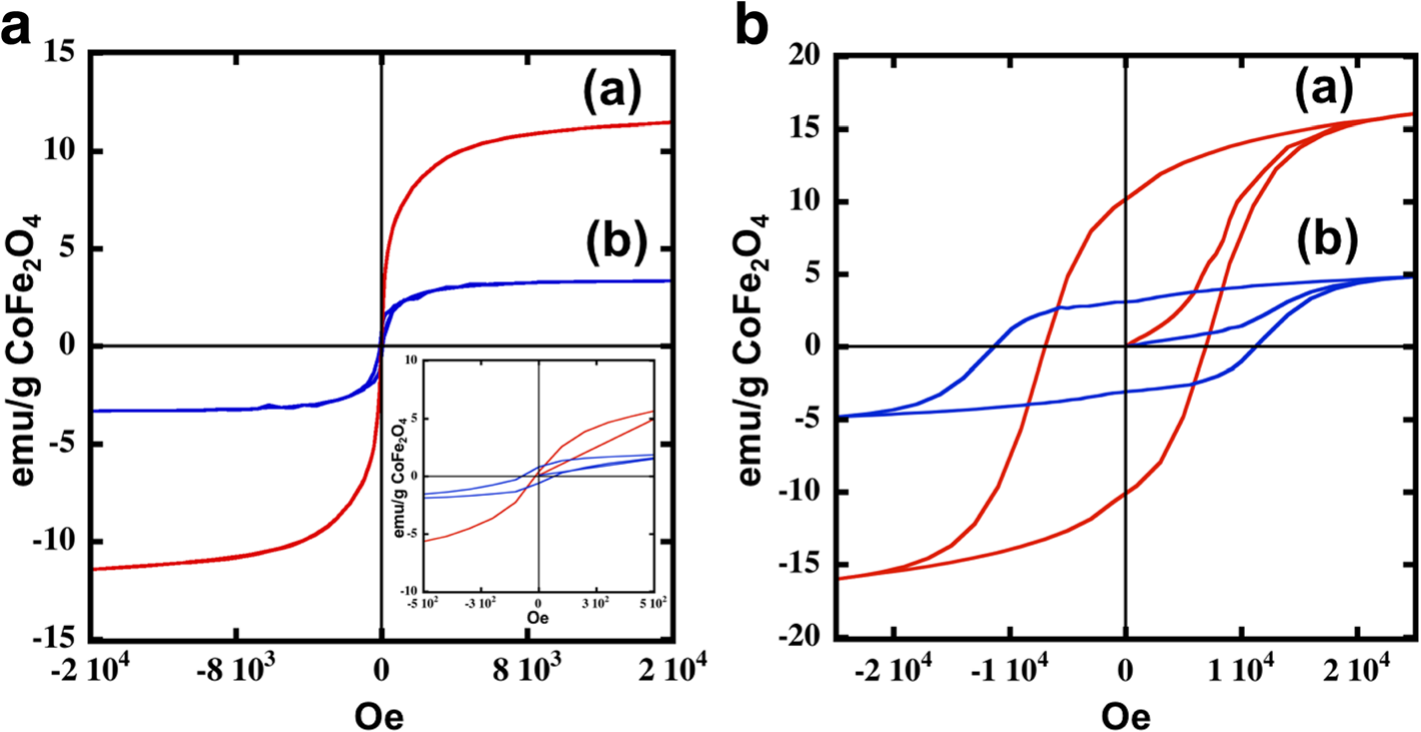
Hysteresis loops for nanoparticles: (**a**) and (**b**) are for the CoFe_2_O_4_ nanoparticles (red line) and CoFe_2_O_4_ @Ag nanoparticles (blue line), respectively, at **a** 300 K and **b** at 5 K

The magnetic hysteresis loops of films made up of the CoFe_2_O_4_ and the CoFe_2_O_4_@Ag nanoparticles were measured at 300 and 5 K, as shown in Fig. 4. These hysteresis loops were normalized as the magnetic susceptibility per unit cobalt weight. Due to the analysis of the crystallographic phase using XRD (Fig. 2), the crystalline densities of CoFe_2_O_4_ and CoFe_2_O_4_@Ag nanoparticles were estimated to be 5.3 and 10.5 g/cm^3^, respectively. Also, the volumes of CoFe_2_O_4_ and CoFe_2_O_4_@Ag nanoparticles were calculated using the results from the TEM observation (Fig. 1). CoFe_2_O_4_ nanoparticles showed a superparamagnetic behavior at room temperature (Fig. 4a). As mentioned by López-Ortega et al. [17], the CoFe_2_O_4_ nanoparticles with the size below 20 nm showed the superparamagnetic behavior at room temperature. The magnetic properties of each sample at the two temperatures are summarized in Table 1. Magnetic saturation (*M*_s_) of the CoFe_2_O_4_ nanoparticles was 11 (emu/g CoFe_2_O_4_), which is lower than the previous results [17, 30, 31]. This is possibly owing to the smaller particle size obtained in this study. On the other hand, the *M*_s_ of the CoFe_2_O_4_@Ag was even smaller with a value of 3.3 (emu/g, CoFe_2_O_4_). As mentioned in the previous literature for Fe_3_O_4_@Ag nanoparticles [8–10, 32–34], the *M*_s_ of CoFe_2_O_4_@Ag decreases possibly due to the diamagnetic contribution of the Ag shell. Moreover, CoFe_2_O_4_@Ag showed 77 Oe, which is high *H*_c_ value at 300 k. The *H*_c_ of the CoFe_2_O_4_@Ag is also different from that of CoFe_2_O_4_ under the low temperature (Fig. 4b). Both of the nanoparticles exhibited ferromagnetism at 5 K despite their relatively small sizes. On the basis of the data near zero magnetization, the value of *H*_c_ increases for CoFe_2_O_4_@Ag nanoparticles (7 k Oe for CoFe_2_O_4_ and 11 k Oe for CoFe_2_O_4_@Ag). This interesting behavior has also been observed in other core-shell nanoparticles such as Fe@Ag [10] and Fe_3_O_4_@Au nanoparticles [5]. Taking these facts into account, the increase of the *H*_c_ of the CoFe_2_O_4_@Ag nanoparticles can be derived from a less-effective coupling of magnetic dipole moment [5, 20].

**Table 1:** Magnetic properties of CoFe_2_O_4_ nanoparticles and CoFe_2_O_4_@Ag nanoparticles

Nanoparticle	*M*_s_ (5 k) (emu/g, CoFe204)	*H*_c_ (5 k) (KOe)	Ms (300 k) (emu/g, CoFe204)	He (300 k) (Oe)
CoFe_2_O_4_	16	7	11	–
CoFe_2_O_4_@Ag	4.8	11	3.3	72

**Fig. 5:**
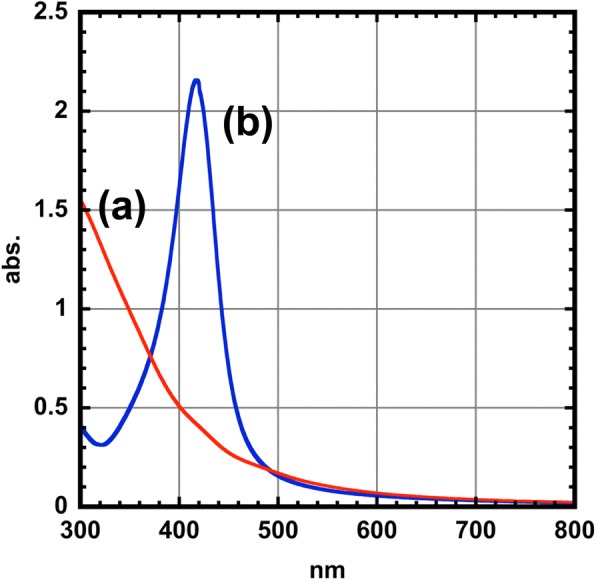
UV-vis spectra for (**a**) CoFe_2_O_4_ nanoparticles (red line) and (**b**) CoFe_2_O_4_ @Ag nanoparticles (blue line)

Next, optical properties of the CoFe_2_O_4_ nanoparticles were investigated by UV-visible spectral measurements. Ag nanoparticles are known to show significant light extinction in the visible region due to the excitation of localized surface plasmon resonance (LSPR) by the coupling of the irradiated light with the coherent oscillation of surface electrons within the Ag nanoparticles. Although the CoFe_2_O_4_ nanoparticles showed no LSPR extinction band in the visible region (Fig. 5), the colloidal solution of our core-shell type CoFe_2_O_4_@Ag nanoparticles showed a sharp extinction peak at 416 nm. This can be attributed to the plasmon absorption (dipole mode) of the Ag shell, which is theoretically supported by the Mie theory (see Additional file 1). This interesting behavior has been observed for Fe@Ag nanoparticles [10] and Co@Ag nanoparticles [7]. In addition, the spectroscopic properties of the CoFe_2_O_4_@Ag nanoparticles were not changed for 1 month, indicating the superior stability of the nanoparticles under air.

**Fig. 6:**
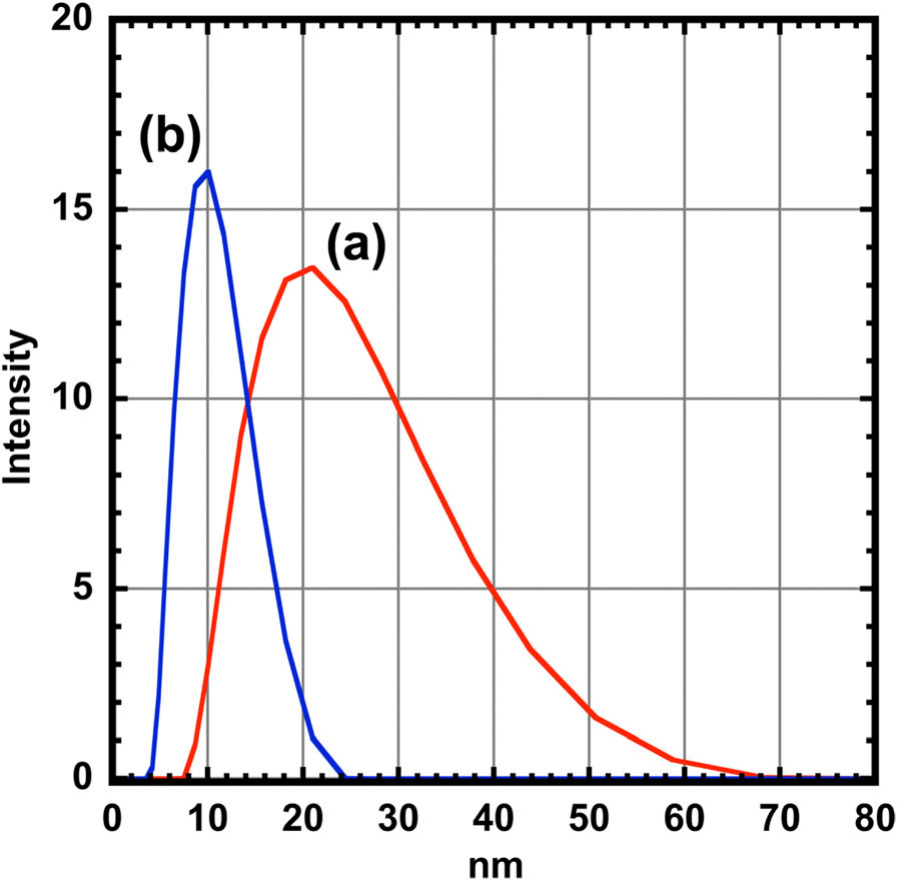
Size distribution (**a**) of the CoFe_2_O_4_ (red line) and (**b**) the CoFe_2_O_4_@Ag nanoparticles (blue line) measured by DLS

The colloidal stability of the CoFe_2_O_4_ and the CoFe_2_O_4_@Ag nanoparticles was evaluated by measuring the size distributions of the nanoparticles in hexane using DLS (Fig. 6). The average sizes of the CoFe_2_O_4_ and CoFe_2_O_4_@Ag nanoparticles were measured to be 19.67 and 9.27 nm, respectively. The sizes of these nanoparticles obtained from TEM, XRD, and DLS measurements are summarized in Table 2. The main difference in sizes measured by these two techniques is due to the presence of an adsorption layer consisting of the OA and OAm on the surface of the particles [35]. Organic compounds such as OA and OAm did not appear in TEM images due to the electron permeability (Fig. 1). Given that the chain lengths of the OA and the OAm are roughly 2 nm [36, 37], the size of CoFe_2_O_4_@Ag estimated by the TEM is slightly (ca. 4 nm) larger than that by the DLS. On the other hand, it is reasonable that the size of CoFe_2_O_4_ by the DLS is far larger than that estimated from this assumption. These results suggest that CoFe2O4 nanoparticles are agglomerated in hexane. This factor includes not only the size effect of the particles described above but also the low affinity between the CoFe_2_O_4_ surfaces and the protective agents. The tendency of agglomeration of the CoFe_2_O_4_ may not only due to the size effect of the particles described above but also due to the low affinity between the CoFe_2_O_4_ surfaces and the protective agents. Precipitation of CoFe_2_O_4_ nanoparticles was observed much more frequently than CoFe_2_O_4_@Ag nanoparticles in the process of redispersion by increasing the number of methanol washing. The high monodispersity of CoFe_2_O_4_@Ag is strongly supported by the low polydispersity index (PDI) obtained by the DLS measurements [38]. These results indicate that the coating with Ag adds not only an optical function but also the stability in solution to the CoFe_2_O_4_ nanoparticles.

**Table 2:** Summary of the sizes of NPs obtained from TEM, XRD, and DLS analysis

Nanoparticle	Size from TEM (nm)	Size from XRD (nm)	Size from DLS (nm) in hexane (PDI)
CoFe_2_O_4_	3.2 ± 0.76	7.1	19.67 (0.153)
CoFe_2_O_4_@Ag	5.3 ± 0.76	3.5	9.27 (0.083)

## Conclusions

The CoFe_2_O_4_@Ag nanoparticles synthesized by a simple and rapid one-pot process were found to be formed on having a uniform core-shell structure with a narrow size distribution from TEM images (Fig. 6). Also, these nanoparticles showed a multifunctionality consisting of the plasmonic light extinction property and a superparamagnetic behavior at room temperature. Furthermore, the core-shell nanoparticles showed higher *H*_c_ than CoFe_2_O_4_ nanoparticles at 5 K and 300 k. In addition, these nanoparticles maintained high monodispersity in an organic solvent. The uniform nanoparticles synthesized by the simple process have a great potential in various fields owing to the multifunctionality as well as the stability.

## Additional File

Additional file1: **Figure S1.** (A) Model of core-shell nanoparticle. Calculated extinction spectra of the core-shell nanoparticle consisting of only the multipolar: (B) and dipolar plasmon modes: (C) surrounded by hexane (*n* = 1.3740). The dielectric value of core is varied: (a) 1, (b) 3, (c) 6, (d) 9, (e) 12, and (f) 15. (DOCX 110 kb)

## Abbreviations

DLS: Dynamic light scattering
fcc: Face-centered cubic
*H*_c_: Coercive field
*M*_s_: Magnetic saturation
OA: Oleic acid
OAm: Oleylamine
PDI: Low polydispersity index
SQUID: Superconducting quantum interference device
TEM: Field-emission transmission electron microscopy
XPS: X-ray photoelectron spectroscopy
XRD: X-ray diffraction

## References

[1] Park HY, Schadt MJ, Wang L, Lim IIS, Njoki PN, Kim SH, Jang MY, Luo J, Zhong CJ (2007) Fabrication of magnetic core @Shell Fe Oxide@ Au nanoparticles for interfacial bioactivity and bio-separation. Langmuir. 23(17):9050–9056. 10.1021/la701305f 10.1021/la701305f 17629315

[2] Wu W, He Q, Jiang C (2008) Magnetic iron oxide nanoparticles: Synthesis and surface functionalization strategies. Nanoscale Res Lett 3(11):397-415. 10.1007/s11671-008-9174-9 10.1007/s11671-008-9174-9 PMC3244954 21749733

[3] Kharisov BI, Dias HVR, Kharissova OV, Vázquez A, Peña Y, Gómez I (2014) Solubilization, dispersion and stabilization of magnetic nanoparticles in water and non-aqueous solvents: recent trends. RSC Adv 4(85):45354-45381. 10.1039/C4RA06902A

[4] Du M, Liu Q, Huang C, Qiu X (2017) One-step synthesis of magnetically recyclable Co@BN core–shell nanocatalysts for catalytic reduction of nitroarenes. RSC Adv 7(56):35451–35459. 10.1039/C7RA04907B

[5] Wang L, Luo J, Fan Q, Suzuki M, Suzuki IS, Engelhard MH, Lin Y, Kim N, Wang JQ, Zhong C-J (2005) Monodispersed core-shell Fe3O4@Au nanoparticles. J Phys Chem B 109(46):21593–21601. 10.1021/jp0543429 10.1021/jp0543429 16853803

[6] Kelly AT, Filgueira CS, Schipper DE, Halas NJ, Whitmire KH (2017) Gold coated iron phosphide core–shell structures. RSC Adv. 7(42):25848–25854. 10.1039/C7RA01195D

[7] Garcia-Torres J, Vallés E, Gómez EJ (2010) Synthesis and characterization of Co@Ag core-shell nanoparticles. J Nanoparticle Res 12(6):2189–2199. 10.1007/s11051-009-9784-x

[8] Xu Z, Hou Y, Sun SJ (2007) Magnetic core/shell Fe3O4/Au and Fe3O 4/Au/Ag nanoparticles with tunable plasmonic properties. J Am Chem Soc 129(28):8698–8699. 10.1021/ja073057v 10.1021/ja073057v 17590000

[9] Mandal M, Kundu S, Ghosh SK, Panigrahi S, Sau TK, Yusuf SM, Pal TJ (2005) Magnetite nanoparticles with tunable gold or silver shell. J Colloid Interface Sci. 286(1):187–194. 10.1016/j.jcis.2005.01.013 10.1016/j.jcis.2005.01.013 15848416

[10] Lu L, Zhang W, Wang D, Xu X, Miao J, Jiang Y (2010) Fe@Ag core-shell nanoparticles with both sensitive plasmonic properties and tunable magnetism. Mater Lett 64(15):1732–1734. 10.1016/j.matlet.2010.04.025

[11] Ferjaoui Z, Schneider R, Meftah A, Gaffet E, Alem H (2017) Functional responsive superparamagnetic core/shell nanoparticles and their drug release properties. RSC Adv 7(42):26243–26249. 10.1039/c7ra02437a

[12] Nairan A, Khan U, Iqbal M, Khan M, Javed K, Riaz S, Naseem S, Han X (2016) Structural and Magnetic Response in Bimetallic Core/Shell Magnetic Nanoparticles. Nanomaterials. 6(4):72. 10.3390/nano6040072 10.3390/nano6040072 PMC5302569 28335200

[13] Song Q, Zhang ZJJ (2004) Shape Control and Associated Magnetic Properties of Spinel Cobalt Ferrite Nanocrystals. J Am Chem Soc 126(19):6164–6168. 10.1021/ja049931r 10.1021/ja049931r 15137781

[14] Tsai C-F, Chen L, Chen A, Khatkhatay F, Zhang W, Wang H (2013) Enhanced Flux Pinning Properties in Self-Assembled Mangetic CoFe2O4 Nanoparticles Doped YBa2Cu3O7−δ Thin Films. IEEE Trans Appl Supercond 23(3):8001204.

[15] Chen D, Yi X, Chen Z, Zhang Y, Chen B, Kang Z (2014) Synthesis of CoFe2O4 nanoparticles by a low temperature microwave-assisted ball-milling technique. Int J Appl Ceram Technol 11(5):954–959. 10.1111/ijac.12110

[16] Chinnasamy CN, Jeyadevan B, Shinoda K, Tohji K, Djayaprawira DJ, Takahashi M, Justin Joseyphus R, Narayanasamy A (2003) Unusually high coercivity and critical single-domain size of nearly monodispersed CoFe2O4 nanoparticles. Appl Phys Lett 83(14):2862–2864. 10.1063/1.1616655

[17] López-Ortega A, Lottini E, Fernández CDJ, Sangregorio C (2015) Exploring the Magnetic Properties of Cobalt-Ferrite Nanoparticles for the Development of a Rare-Earth-Free Permanent Magnet. Chem Mater 27(11):4048–4056. 10.1021/acs.chemmater.5b01034

[18] Sun S, Zeng H, Robinson DB, Raoux S, Rice PM, Wang SX, Li GJ (2004) Monodisperse MFe 2 O 4 (M = Fe, Co, Mn) Nanoparticles. J Am Chem Soc 126(1):273–279. 10.1021/ja0380852 10.1021/ja0380852 14709092

[19] Song Y, Ding J, Wang Y (2012) Shell-dependent evolution of optical and magnetic properties of Co@Au core-shell nanoparticles. J Phys Chem C 116(20):11343–11350. 10.1021/jp300118z

[20] Wang L, Park H-Y, Lim SI-I, Schadt MJ, Mott D, Luo J, Wang X, Zhong C-JJ (2008) Core@shell nanomaterials: gold-coated magnetic oxide nanoparticles. J Mater Chem 18(23):2629. 10.1039/b719096d

[21] Sharma SK, Vargas JM, Vargas NM, Castillo-Sepúlveda S, Altbir D, Pirota KR, Zboril R, Zoppellaro G, Knobel MR (2015) Unusual magnetic damping effect in a silver–cobalt ferrite hetero nano-system. R Soc Chem Adv 5:17117–17122. 10.1039/C4RA14960B

[22] Kooti M, Saiahi S, Motamedi HJ (2013) Fabrication of silver-coated cobalt ferrite nanocomposite and the study of its antibacterial activity. J Magn Magn Mater 333:138–143. 10.1016/j.jmmm.2012.12.038

[23] Zhang K, Xiang Y, Wu X, Feng L, He W, Liu J, Zhou W, Xie S. (2009) Enhanced Optical Responses of Au @ Pd Core / Shell Nanobars 8:1162–1168 10.1021/la803060p 19090666

[24] Mätzler, C (2002) MATLAB Functions for Mie Scattering and Absorption. IAP Res Rep 2002-08(July 2002):1139–1151. 10.1039/b811392k

[25] Ordal MA, Bell, R. J, Alexander RW, Long LL, Querry MR (1985) Optical properties of fourteen metals in the infrared and far infrared: Al, Co, Cu, Au, Fe, Pb, Mo, Ni, Pd, Pt, Ag, Ti, V, and W. 24(24):4493–4499 10.1364/ao.24.004493 18224235

[26] Wu W, Wu Z, Yu T, Jiang C, Kim WS (2015) Recent progress on magnetic iron oxide nanoparticles: Synthesis, surface functional strategies and biomedical applications. Sci Technol Adv Mater. 16(2). 10.1088/1468-6996/16/2/023501 10.1088/1468-6996/16/2/023501 PMC5036481 27877761

[27] Stefan M, Leostean C, Pana O, Soran ML, Suciu RC, Gautron E, Chauvet O (2014) Synthesis and characterization of Fe3O4@ZnS and Fe3O4@Au@ZnS core-shell nanoparticles. Appl Surf Sci 288:180–192. 10.1016/j.apsusc.2013.10.005

[28] Tang R, Jiang C, Qian W, Jian J, Zhang X, Wang H, Yang H (2015) Dielectric relaxation, resonance and scaling behaviors in Sr3Co2Fe24O41 hexaferrite. Sci Rep 5:1–11. 10.1038/srep13645 10.1038/srep13645 PMC4551993 26314913

[29] Sun Y, Ji G, Zheng M, Chang X, Li S, Zhang YJ (2010) Synthesis and magnetic properties of crystalline mesoporous CoFe 2 O 4 with large specific surface area. J Mater Chem 20(5):945–952. 10.1039/B919090B

[30] Bohara RA, Thorat ND, Yadav HM, Pawar SH (2014) One-step synthesis of uniform and biocompatible amine functionalized cobalt ferrite nanoparticles: a potential carrier for biomedical applications. New J Chem 38(7):2979. 10.1039/c4nj00344f

[31] Pervaiz E, Humaira IHG (2015) Hydrothermal Synthesis and Characterization of CoFe 2 O 4 Nanoparticles and Nanorods. J Appl Phys 117. 10.1007/s10948-012-1749-0

[32] Park J, Lee E, Hwang N-M, Kang M, Kim SC, Hwang Y, Park J-G, Noh H-J, Kim J-Y, Park J-H, Hyeon T (2005) One-Nanometer-Scale Size-Controlled Synthesis of Monodisperse Magnetic Iron Oxide Nanoparticles. Angew Chemie Int Ed 44(19):2872–2877. 10.1002/anie.200461665 10.1002/anie.200461665 15798989

[33] Wang C, Xu J, Wang J, Rong Z, Li P, Xiao R, Wang S (2015) Polyethylenimine-interlayered silver-shell magnetic-core microspheres as multifunctional SERS substrates. J Mater Chem C 3(33):8684–8693. 10.1039/C5TC01839K

[34] Walker JM, Zaleski JM (2016) A simple route to diverse noble metal-decorated iron oxide nanoparticles for catalysis. Nanoscale 8(3):1535–1544. 10.1039/C5NR06700F 10.1039/c5nr06700f 26681072

[35] Lim J, Yeap S, Che H, Low S (2013) Characterization of magnetic nanoparticle by dynamic light scattering. Nanoscale Res Lett 8(1):381. 10.1186/1556-276X-8-381 10.1186/1556-276X-8-381 PMC3846652 24011350

[36] Wang Z, Wen X-D, Hoffmann R, Son JS, Li R, Fang C-C, Smilgies D-M, Hyeon T (2010) Reconstructing a solid-solid phase transformation pathway in CdSe nanosheets with associated soft ligands. Proc Natl Acad Sci 107(40):17119–17124. 10.1073/pnas.1011224107 10.1073/pnas.1011224107 PMC2951424 20855580

[37] Zhang L, He R, Gu HC (2006) Oleic acid coating on the monodisperse magnetite nanoparticles. Appl Surf Sci 253(5):2611–2617. 10.1016/j.apsusc.2006.05.023

[38] Araújo-Neto RP, Silva-Freitas EL, Carvalho JF, Pontes TRF, Silva KL, Damasceno IHM, Egito EST, Dantas AL, Morales MA, Carriço ASJ (2014) Monodisperse sodium oleate coated magnetite high susceptibility nanoparticles for hyperthermia applications. J Magn Magn Mater 364:72–79. 10.1016/j.jmmm.2014.04.001

