# Supplementary material for: One-pot synthesis of monodisperse CoFe2O4@Ag core-shell nanoparticles and their characterization
Source: Nanoscale Res Lett. 2018 Jun 8;13:176. doi: 10.1186/s11671-018-2544-z (PMC5993709; doi:10.1186/s11671-018-2544-z)
Supplement: Supplementary file 1 — Figure S1. (A) Model of core-shell nanoparticle. Calculated extinction spectra of the core-shell nanoparticle consisting of only the multipolar: (B) and dipolar plasmon modes: (C) surrounded by hexane (n = 1.3740). The dielectric value of core is varied: (a) 1, (b) 3, (c) 6, (d) 9, (e) 12, and (f) 15. (DOCX 110 kb) [file 11671_2018_2544_MOESM1_ESM.docx]

*Supporting Information for*

**One-pot synthesis of monodisperse CoFe_2_O_4_@Ag core-shell**

**nanoparticles and their characterization**

Shuta Hara,^1^ Junpei Aisu,^1^ Masahiro Kato,^1^ Takashige Aono,^2^ Kosuke Sugawa,^1^ Kouichi Takase,^2^ Joe Otsuki,^1^ Shigeru Shimizu,^1^ Hiroki Ikake*^1^

1. Characterization of optical properties of CoFe_2_O_4_@Ag nanoparticles

Using a model on the basis of the TEM observation of our synthesized core-shell CoFe_2_O_4_@Ag nanoparticles (Fig. S1(A)), we calculated optical properties of the core-shell nanoparticles. Figs. S1(B) and (C) show calculated extinction spectra consisting of multipolar plasmon modes and only the dipolar plasmon mode, respectively. Such a core-shell type nanoparticle has a sharp extinction peak at a visible region, which is the same as the experimentally-obtained spectrum (Fig. 4 in the manuscript). Also, although moderate damping and red-shifting of the peak occurs with increasing the dielectric constant of the core, the sharp spectral shape is maintained. Furthermore, the spectral shape of the multipolar plasmon modes is almost the same as that of only the dipolar plasmon mode. These results suggest that the dipole plasmon mode is dominant in the core-shell type nanoparticles, although the dielectric function of CoFe_2_O_4_ core within the visible region is unclear.


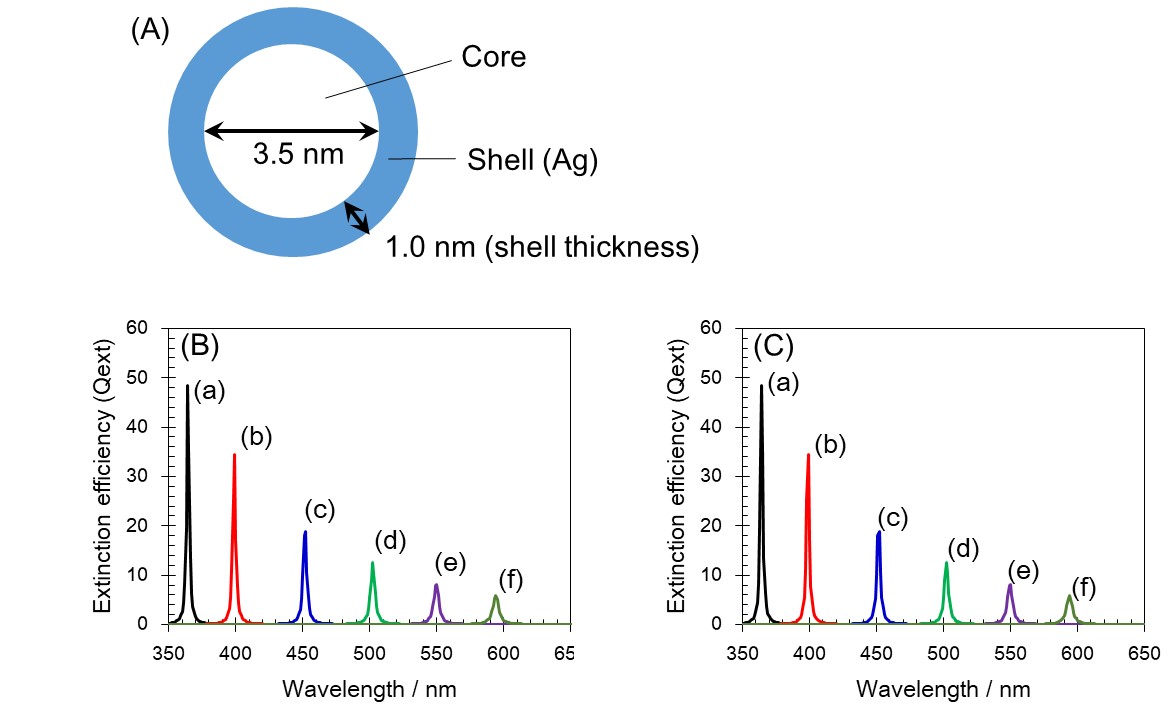


Figure S1. (A) Model of core-shell nanoparticle. Calculated extinction spectra of the core-shell nanoparticle consisting of only the multipolar: (B) and dipolar plasmon modes: (C) surrounded by hexane (*n* = 1.3740). The dielectric value of core is varied: (a) 1, (b) 3, (c) 6, (d) 9, (e) 12 and (f) 15.
